# Avoiding methodological bias in studies of amyloid imaging results disclosure

**DOI:** 10.1186/s13195-019-0495-y

**Published:** 2019-06-04

**Authors:** Carl Taswell, Cheryl Donohue, Maree T. Mastwyk, Andrea G. Louey, Jacqueline Giummarra, Joanne Robertson, David G. Darby, Colin L. Masters, Christopher C. Rowe

**Affiliations:** 1 Brain Health Alliance, Ladera Ranch, CA USA; 2 Department Psychiatry, University California San Diego, La Jolla, CA USA; 3 Florey Institute Neuroscience and Mental Health, University Melbourne, Parkville, VIC Australia; 4 Austin Health Department Molecular Imaging, Melbourne, VIC Australia

We read with interest the paper by Grill et al. [1], a study of *N* = 33 participants interviewed about their reactions to learning that their amyloid imaging results were “not elevated” in a preclinical Alzheimer’s disease (AD) trial. We welcome their contribution to the literature and would like to call attention to some related larger-size studies by Wake et al. [2] (with *N* = 42 subjects) and by Taswell et al. [3] (with *N* = 133 subjects) that were not cited in the paper by Grill et al. [1].

Neurodegenerative cognitive disorders, including AD, remain challenging to elucidate pathophysiologically and to prevent or treat pharmacologically. Clinicians who study psychological and behavioral health disorders (including those without cognitive decline) may readily argue that it is even more challenging to understand and predict human behavior. Subjective interviews and objective psychometrics using questionnaires completed by research subjects and/or by trained observers cannot be performed or interpreted with any guarantees of infallible reliability for predictions about future behavior. As perhaps the most dramatic example, experienced clinicians in emergency psychiatry recognize and appreciate the critical limitations of both subjective interviews and objective psychometrics when performing suicide risk assessments (Range et al. [4] and Erford et al. [5]) for patients evaluated per mental healthcare statutes for possible involuntary hospitalization and a denial of rights to leave the locked care facility.

Due to the known deficiencies of such suicide risk assessments, clinicians in this scenario compensate by relying on a multiplicity of different approaches with different observers, reporters, questionnaires, and tools for the evaluation of the patient in an effort to improve the overall quality (validity and reliability) of the clinical evaluation and risk assessment, thus hopefully reducing the probability of the worst-case outcome, the patient’s death by suicide. Noble et al. [6] described this methodology in general as “data triangulation, whereby different methods and perspectives help produce a more comprehensive set of findings.”

In the large-size study with *N* = 133 subjects that we reported on disclosure of amyloid imaging results to patients with mild cognitive impairment (MCI) and early AD (Taswell et al. [3]), we used this approach with multiple comparison groups (amyloid negative versus amyloid positive, MCI versus AD, younger *<* 70 versus older *≥* 70), a diverse collection of psychometric questionnaires, as well as observable outcomes that were reportable life events. In particular, we described in our findings that there were “no concerns expressed by any patients, family or caregivers about any real potential risk of harm to patients such as reports of suicidal ideation threats or plans, with or without visits to doctors’ offices, psychiatric emergency rooms or hospitals for any such complaints.” As a consequence, we maintain high confidence in our conclusion that “we consider [ed] it safe, without apparent risk of harm to patients, to disclose amyloid imaging results to patients who have no prior history of neuropsychiatric illness.”

Methodological bias in a research study can be avoided when there is no selection bias on the subjects, no investigator bias on the examinations (or interviews or psychometrics), no bias with the tools used, and no absence of comparison groups, etc. If we have a greater number of methodological approaches in the clinical trial design that counter possible methodological biases, then we can interpret the clinical trial results with greater confidence. If a diverse collection of examination tools yields the same consistent results, then it is more likely that those results are true. While it may be difficult to pursue an ideal clinical trial, we should nevertheless aspire to conduct trials with a larger sample size of subjects, a diversity of examiners and/or examination tools, and hopefully two or more comparison groups so that we can make some kind of comparison. Although we note that the authors of Grill et al. [1] discussed some limitations of their clinical trial, they did not discuss the absence of a comparison group in their study.

Thus, we encourage Grill et al. [1] to investigate in their next study of disclosing amyloid imaging results that are *not elevated* also the comparison group of participants with amyloid imaging results that are *elevated*. Alternatively, in the absence of two or more comparison groups, each subject can always be compared to self if the subject has been examined at serial time points with pre- and post-disclosure interviews and/or psychometric exams that permit calculation of individual subject change scores (*after* versus *before* disclosure) as done in our study by Taswell et al. [3]. Nevertheless, having two or more comparison groups in an amyloid imaging result disclosure study, such as comparing participants from the same AD pre-clinical trial who have either *elevated* or *not elevated* amyloid (even if the study has small sample size of insufficient power with other limitations), would nevertheless hopefully yield some basic intuition and anecdotal experience with a comparison between the two different groups of subjects when they participate in the interviews and psychometrics. Are they the same or different? Or are the investigators unable to answer that question of *same versus different* because of other limitations introduced by methodological biases inherent in the study design?

Readers interested in learning more about the pitfalls of methodological bias in clinical trial design and research should consult the important body of literature available on this topic (Schulz [7], Smyth et al. [8], Higgins et al. [9], Hrobjartsson et al. [10], Kirkham et al. [11]). In particular, Weuve et al. [12] provided some guidelines for evaluating potential bias in dementia research. Clinical trial investigators should also keep in mind the following important principle: the higher the quality of the study design without methodological bias, the greater the probability that results from the study can be considered for inclusion in a systematic review and meta-analysis of the medical-scientific question addressed. We strongly recommend consideration of clinical trial designs that avoid methodological bias with a multiplicity of examiners and/or examination tools, a multiplicity of comparison groups, inclusion of observable life events in addition to interviews or psychometrics as outcome measures, and larger rather than smaller sample size whenever possible.
